# Iron trapping in macrophages reshapes the homeostasis of the haematopoietic system

**DOI:** 10.1111/bjh.20031

**Published:** 2025-02-26

**Authors:** Laura Crisafulli, Margherita Correnti, Elena Gammella, Elisa De Camilli, Matteo Brindisi, Eleonora Palagano, Chiara Milanesi, Gabriele Todisco, Matteo G. Della Porta, Cristina Sobacchi, Gaetano Cairo, Francesca Ficara, Stefania Recalcati

**Affiliations:** ^1^ Milan Unit CNR‐IRGB Milan Italy; ^2^ IRCCS Humanitas Research Hospital Milan Italy; ^3^ Department of Biomedical Sciences for Health University of Milan Milan Italy; ^4^ Division of Pathology, IEO European Institute of Oncology IRCCS Milan Italy; ^5^ Institute of Biosciences and Bioresources CNR Florence Italy; ^6^ Department of Biomedical Sciences Humanitas University Pieve Emanuele Italy

**Keywords:** anaemia, animal model, haematopoiesis, iron, macrophages, red cells

## Abstract

Iron is required for key physiological processes, like oxygen transport, energy production and cell proliferation. Body iron homeostasis is regulated by the erythroferrone–hepcidin–ferroportin (FPN) axis, which mainly acts on absorptive duodenal cells and macrophages involved in iron recycling from red blood cell breakdown. In addition to systemic iron regulation, macrophages are also involved in local iron release to neighbouring cells. Similarly, bone marrow (BM)‐resident macrophages could represent promptly available local sources of iron for developing haematopoietic cells. To study the impact of macrophage‐released iron on BM haematopoietic stem and progenitor cells, we employed mice with targeted deletion of *Fpn* in the myeloid lineage (*Fpn* conditional knockout or *Fpn*‐cKO). *Fpn*‐cKO mice develop age‐related anaemia and microcytaemia, reduction of BM erythroblasts and preferential megakaryopoiesis at the expenses of erythropoiesis, suggesting that red cells are mostly affected by the lack of myeloid‐derived iron delivery. Transferrin receptor 1 surface expression is higher in *Fpn*‐cKO mice than littermate controls in all the BM subpopulation analysed, starting from haematopoietic stem cells, indicating a broad BM sensitivity to lower iron availability. Last, *Fpn*‐cKO mice activate systemic compensatory mechanisms, such as extramedullary haematopoiesis and erythroferrone upregulation, albeit not sufficient to overcome anaemia.

## Graphical abstract

Deletion of ferroportin (*Fpn*) in myeloid cells results in iron trapping in macrophages and reshapes whole haematopoiesis with ageing. Impaired local iron supply to bone marrow (BM) leads to anaemia and microcytaemia, unbalanced megakaryopoiesis versus erythropoiesis and reduced BM cells starting from haematopoietic stem cells. Several systemic and local mechanisms are activated to compensate the chronic BM dysfunction, including extramedullary haematopoiesis and enhanced BM iron avidity through an increased membrane expression of transferrin receptor 1 (TFR1). However, these attempts are not sufficient to overcome anaemia, highlighting the importance of macrophage‐dependent iron control.

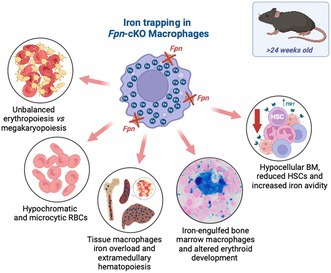

## INTRODUCTION

Iron is required for key physiological processes, such as oxygen transport, energy production and cell proliferation.^1^ Body iron homeostasis is primarily regulated by the erythroferrone (ERFE)–hepcidin–ferroportin (FPN) axis.^1^ Hepcidin binding leads to internalization and degradation of FPN, the only known mammalian iron exporter, thereby inhibiting iron release, particularly from iron‐absorbing duodenal cells and macrophages that recycle iron from red blood cell (RBC) breakdown, which provides 80% of the iron required by erythroblasts.^2^ Increased iron needs for higher haemoglobin (HGB) synthesis and erythroid cell proliferation are met by inhibiting hepcidin expression, enhancing FPN activity and iron availability. In these settings, hepcidin is downregulated by ERFE, synthesized by bone marrow (BM) erythroblasts in response to erythropoietin,^3^ and/or by the hepatokine FGL1.^4^ Conversely, hepcidin induction by elevated iron levels or inflammatory mediators leads to the decreased levels of circulating iron and lower transferrin saturation.^2^

In addition to their role in systemic iron regulation, macrophages release iron also locally to neighbouring cells, representing a promptly available source of iron for the needs of specific niches. In fact, we showed that a steady supply of iron released by macrophage FPN is essential for tissue homeostasis in at least two conditions, follicular development and skin wound healing, which share many similarities, including fast cell growth rate.^5^ We asked whether this occurs also in the haematopoietic system, a known iron‐dependent system necessitating multiple levels of regulation for responding to acute requirements while keeping the capacity to maintain steady state. Within the BM, erythroblastic islands (EI), formed by a ‘nurse’ central macrophage surrounded by erythroid progenitors, provide an environmental niche for RBC production. EI macrophages (EIM) adhere to erythroblasts, favour their proliferation and differentiation and phagocytose extruded nuclei.^6^ Moreover, EIM have been implicated in haem degradation and iron recycling, similar to spleen macrophages.^6^, ^7^ Therefore, EIM, which express the high levels of FPN,^8^ are supposed to contribute to supplying iron to the erythroblasts. Besides EIM, a novel subtype of BM macrophages has been recently described as part of the haematopoietic stem cell (HSC) niche.^9^ However, whether HSC‐niche macrophages deliver iron to HSCs is not known. EIM or other BM‐resident macrophages could be an immediately accessible local source of iron for haematopoietic progenitors and precursors. Indeed, activation of the iron response has been shown to sustain regenerating haematopoietic stem and progenitor cells.^10^ Of note, mild anaemia was detected in 3‐week‐old mice with *Fpn* deletion in the myeloid lineage, accompanied by iron accumulation in macrophages, including BM macrophages.^5^, ^11^ Anaemia disappeared in young adult (12‐week‐old) animals, but whether the absence of FPN in myeloid cells has consequences that become evident over time was not analysed.

In this study, we dissected the long‐term impact of iron trapping in macrophages on BM haematopoietic stem and progenitor cells and BM precursors, including erythroblasts. We found that persistent FPN absence in myeloid cells results in multiple defects in the haematopoietic system, which appear with ageing, including anaemia, thrombocytosis, hypocellular BM and extramedullary haematopoiesis.

## MATERIALS AND METHODS

### Animals

*Fpn* ^fl/fl^ *LysCre* ^+/−^ mice (named *Fpn*‐cKO for *Fpn* conditional knockout)^5^ were cohoused with floxed control littermates (*Fpn* ^fl/fl^ *LysCre* ^−^) in individually ventilated cages in a pathogen‐free animal facility at L.I.T.A., University of Milan. *Fpn*‐cKO mice were born in Mendelian ratios and displayed normal fertility. The efficacy of the CRE‐dependent *Fpn* loss in macrophages was previously demonstrated.^5^ Mice were maintained on a normal diet (157 ppm iron content) until 24 weeks of age or longer (24.6–33.0 weeks, average: 27.8). The study was performed in accordance with the guidelines of the Helsinki declaration and approved by the animal welfare office of the Italian Health Ministry (Authorization n° 250/2020‐PR).

### Flow cytometry

BM cells were harvested by flushing of one lower limb (femur and tibia) and stained as detailed in the Supporting Information.

### Histology

Liver and spleen were processed for paraffin blocks embedding; 4‐μm‐thick sections were stained with haematoxylin/eosin. For iron detection, sections were stained with Perls' Prussian blue. Extramedullary haematopoiesis and iron scores were calculated as detailed in the Supporting Information.

### Statistical analysis

Unpaired Student *t*‐test or Mann–Whitney test was used according to the normal or not‐normal distribution of the data respectively. *p* < 0.05 was considered statistically significant—*p < 0.05; **p < 0.01; ***p < 0.001; ****p < 0.0001. Analyses were performed with GraphPad Software.

## RESULTS

### The absence of iron release from myeloid cells affects selected blood parameters and impacts iron distribution

To dissect the long‐term impact of preventing iron release from myeloid cells on blood and BM, mice with lysozyme (*LysM*)‐Cre‐dependent loss of *Fpn* in myeloid cells (*Fpn*‐cKO hereafter) and their littermate controls (*Fpn* ^fl/fl^ *LysCre* ^−/−^, WT for wild‐type hereafter) were aged at least until 6 months, weighted and then sacrificed to collect blood, serum, BM, liver and spleen. Separate analysis performed for male and female mice provided similar results (not shown); therefore, all experimental groups are representative of both sexes.

**FIGURE 1:**
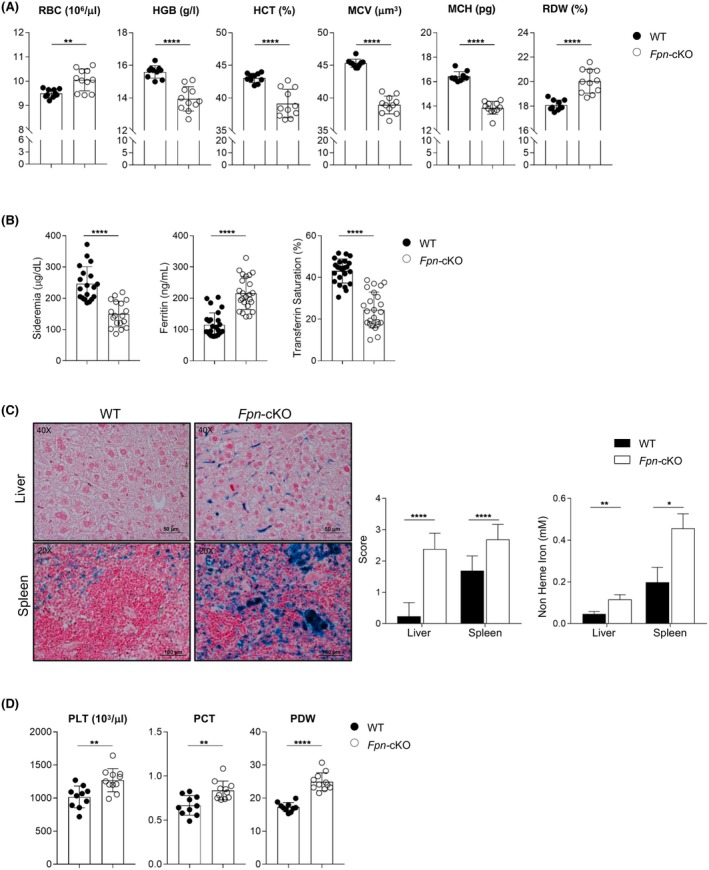
Haematological parameters and iron distribution of *Fpn*‐cKO mice. (A) Blood analysis of erythropoietic parameters. From left to right: Red blood cell (RBC) counts, haemoglobin (HGB) level, haematocrit (HCT), mean corpuscular volume (MCV), mean corpuscular haemoglobin (MCH), red cell distribution width (RDW). (B) Levels of serum iron, ferritin and transferrin saturation. (C) Left: representative histology of Perls' Prussian Blue iron staining of liver and spleen in WT and *Fpn*‐cKO mice. Centre: semiquantitative evaluation of Perls' iron staining. Right: quantification of non‐haem iron in liver and spleen, normalized for the total protein content. (D) Haemocytometric parameters relative to platelets (PLT). From left to right: PLT counts, plateletcrit (PTC), PLT distribution width (PDW). (A, D) WT: *n* = 10 (6 females and 4 males); *Fpn*‐cKO: *n* = 11 (5 females and 6 males); (B) WT: *n* = 19, *Fpn*‐cKO: *n* = 18; (C) For the score, WT: *n* = 16–17 (for spleen and liver respectively), *Fpn*‐cKO: *n* = 13; for non‐haem iron: WT: *n* = 6, *Fpn*‐cKO: *n* = 9. Data are presented as mean ± SD. *p < 0.05; **p < 0.01; ****p < 0.0001.

Blood analysis revealed that most erythropoietic parameters were altered in 6‐month‐old or older (range: 24.6–33 weeks) *Fpn*‐cKO mice, which showed higher RBC counts despite significantly lower haematocrit and HGB levels (Figure 1A) resulting in RBC hypochromia and anisopoikilocytosis (Figure S1A). Accordingly, we observed reduced mean corpuscular haemoglobin and increased RBC distribution width in *Fpn*‐cKO mice; these alterations, coupled with a smaller erythrocyte volume, are indicative of microcytaemia and iron deficiency anaemia. In line with the mild anaemia, serum iron levels were lower in *Fpn*‐cKO mice compared to WT littermates (Figure 1B), as it occurs in iron deficiency anaemia and in anaemia of chronic disease (ACD). We detected higher ferritin levels, coupled with a lower transferrin saturation and iron accumulation in the liver and spleen, as revealed by Perls' Prussian blue iron staining and quantification of non‐haem iron (Figure 1C). These features are usually associated with ACD, in which macrophage iron sequestration is one of the most important pathogenetic mechanisms with consequent reduced iron availability. *Fpn*‐cKO mice also displayed thrombocytosis, with significantly higher platelet (PLT) counts, higher plateletcrit and PLT distribution width (Figure 1D). Accordingly, BM histology revealed an increased number of megakaryocytes (Mk) in *Fpn*‐cKO mice compared to WT (Figure S1B), indicative of enhanced megakaryopoiesis. Total white blood cell (WBC) counts were similar in *Fpn*‐cKO mice and their control littermates, with no differences within WBC subpopulations (lymphocytes, monocytes and granulocytes), neither in absolute counts nor in percentages (Figure S2), suggesting that proper iron availability is mostly relevant for other highly iron‐demanding cells.

The decreased HGB and haematocrit levels, but increased number of RBCs, might indicate higher erythropoietic activity in *Fpn*‐cKO mice, albeit insufficient to restore normal HGB levels.

### Defective iron release from myeloid cells perturbs erythroid development

To gain insight into the peripheral erythropoietic alterations, we analysed the major erythropoietic organs in *Fpn*‐cKO mice.

**FIGURE 2:**
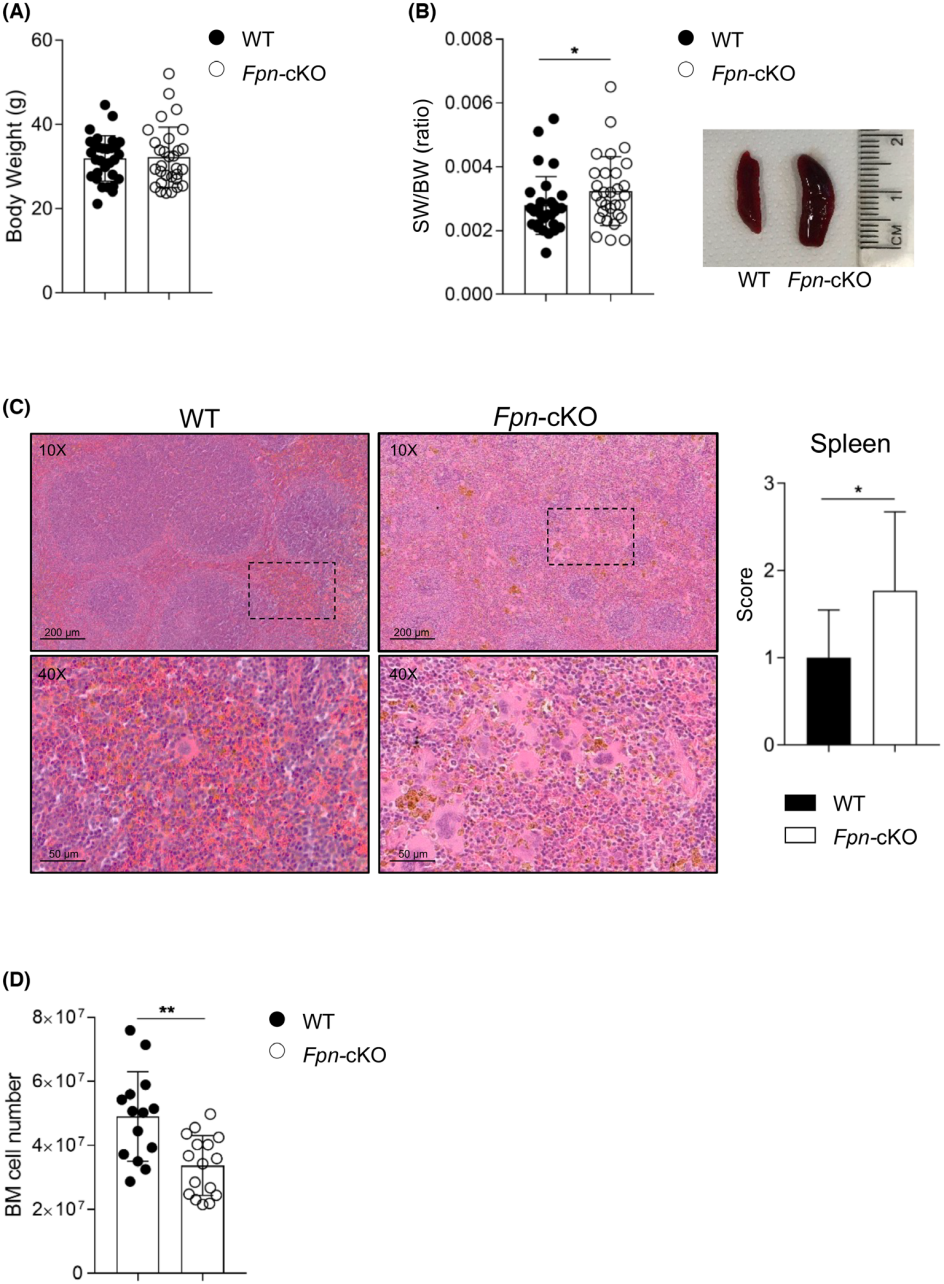
Bone Marrow and Spleen analysis of *Fpn*‐cKO mice. (A) Body weight (BW) of WT and *Fpn*‐cKO mice. (B) Ratio of spleen weight over BW (left) and representative image of spleen size (right). (C) Left: representative images of spleen histology (H&E staining) showing perturbed architecture and myeloid extramedullary haematopoiesis in *Fpn*‐cKO mice. Bottom images depict higher magnification of the indicated inset in the corresponding top images. Right: spleen extramedullary haematopoiesis score. (D) BM cell number (from one lower limb). (A, B) WT: *n* = 30, *Fpn*‐cKO: *n* = 30; (C) WT: *n* = 16, *Fpn*‐cKO: *n* = 13. (D) WT: *n* = 14; *Fpn*‐cKO: *n* = 16. Data are presented as mean ± SD. *p < 0.05; **p < 0.01.

Despite similar body weight, spleen weight was higher in *Fpn*‐cKO mice, with the presence of extramedullary haematopoiesis (Figure 2A–C) which might account for the observed increase in RBC, as an attempt to counteract peripheral anaemia; in line with this finding, the BM was hypocellular (Figure 2D). Altogether, these observations are indicative of possible ineffective BM haematopoiesis.

**FIGURE 3:**
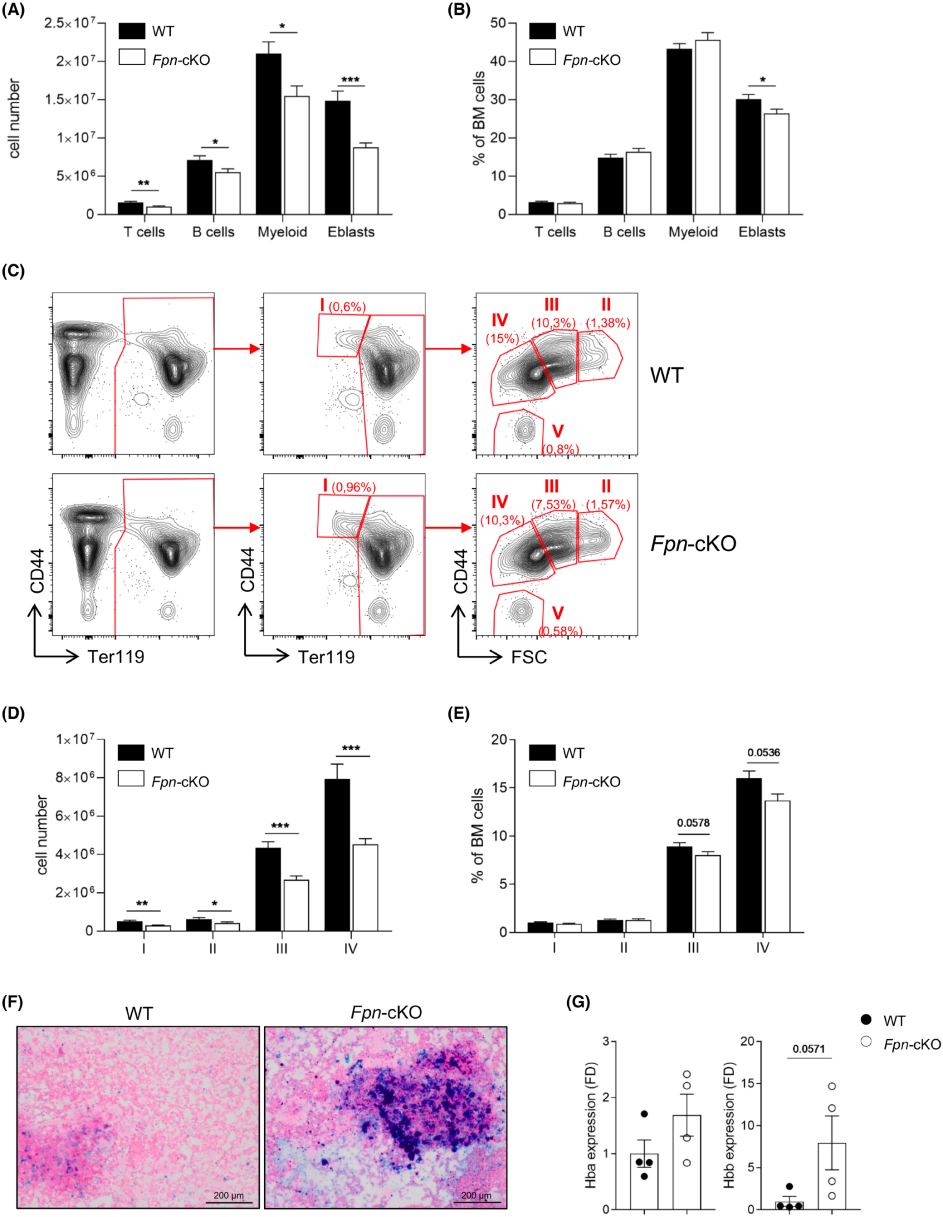
FACS analysis of bone marrow (BM) erythroid development in *Fpn*‐cKO and WT mice. (A) Histogram indicating the absolute number of BM T (CD3^+^) and B (CD19^+^) lymphocytes, myeloid cells (CD11b^+^) and erythroblasts (Eblasts, TER119^+^); data are presented as mean ± SEM. (B) Histogram indicating the frequency of the same cell population depicted in (A). (C) Representative FACS analysis of BM red cell development. (D) Histogram indicating the absolute number of erythroid cells (all TER119^+^) classified in the I–IV progressively more mature fractions, as depicted in (C). (E) Histogram indicating the frequency of the erythroid cells depicted in (D). (F) Representative Perls' Prussian Blue iron staining of BM films in WT and *Fpn*‐cKO mice. (G) Expression of alpha (Hba) and beta (Hbb) globin in sorted BM TER119^+^ erythroblasts measured by quantitative RT‐PCR, normalized to the housekeeping gene 18S RNA and represented as fold difference (FD) of *Fpn*‐cKO mice over WT. BM cells were harvested from one lower limb. WT: *n* = 14; *Fpn*‐cKO: *n* = 16. Data are presented as mean ± SEM. *p < 0.05; **p < 0.01; ***p < 0.001.

To investigate which cell population was mainly responsible for the hypocellular BM, we analysed the frequency of the most abundant BM cell types (lymphocytes, myeloid cells and erythroblasts) by fluorescence‐activated cell sorting (FACS) and calculated their absolute numbers (Figure 3A,B). This analysis revealed that all main BM populations were reduced in cell number; however, most of the reduction was accounted for by decreased erythroblasts, suggesting that these cells are those mostly affected by the lack of iron released by myeloid cells. To further analyse the cause of the erythroblast reduction, we dissected erythroid development by FACS (Figure 3C). All the CD44^+^ erythroid fractions indicative of the different developmental steps, from less mature (fraction I) to orthochromatic erythroblasts (fraction IV), were reduced when considering absolute cell numbers (Figure 3D). Fractions III and IV, which are the relatively most abundant in WT mice, also showed a tendency to reduced frequency within total BM in *Fpn*‐cKO mice compared to WT (Figure 3E). Indeed, despite iron is highly abundant in the BM of *Fpn*‐cKO mice (Figure 3F), it is not available for correct erythroblast development. Therefore, diminished rather than increased BM erythropoiesis likely accounts for the observed peripheral anaemia (i.e. lower HGB and haematocrit). Alternatively, the observed reductions might reflect increased consumption of erythroid progenitors and precursors as an attempt to counteract peripheral anaemia. Together with extramedullary haematopoiesis, this might explain the higher number of RBC, in accordance with the increased expression of alpha and beta globin by erythroblasts from *Fpn*‐cKO mice compared to WT (Figure 3G). However, the increased erythropoiesis is likely not sufficient to ensure the normal amount and frequency of the different cell types within the BM.

### *Fpn*‐cKO mice show alterations in BM and spleen macrophages

Since *Fpn* was deleted in *LysM* ^+^ cells, we then analysed BM myeloid cells to evaluate if iron accumulation could impact their own development and relative abundance in the BM.

**FIGURE 4:**
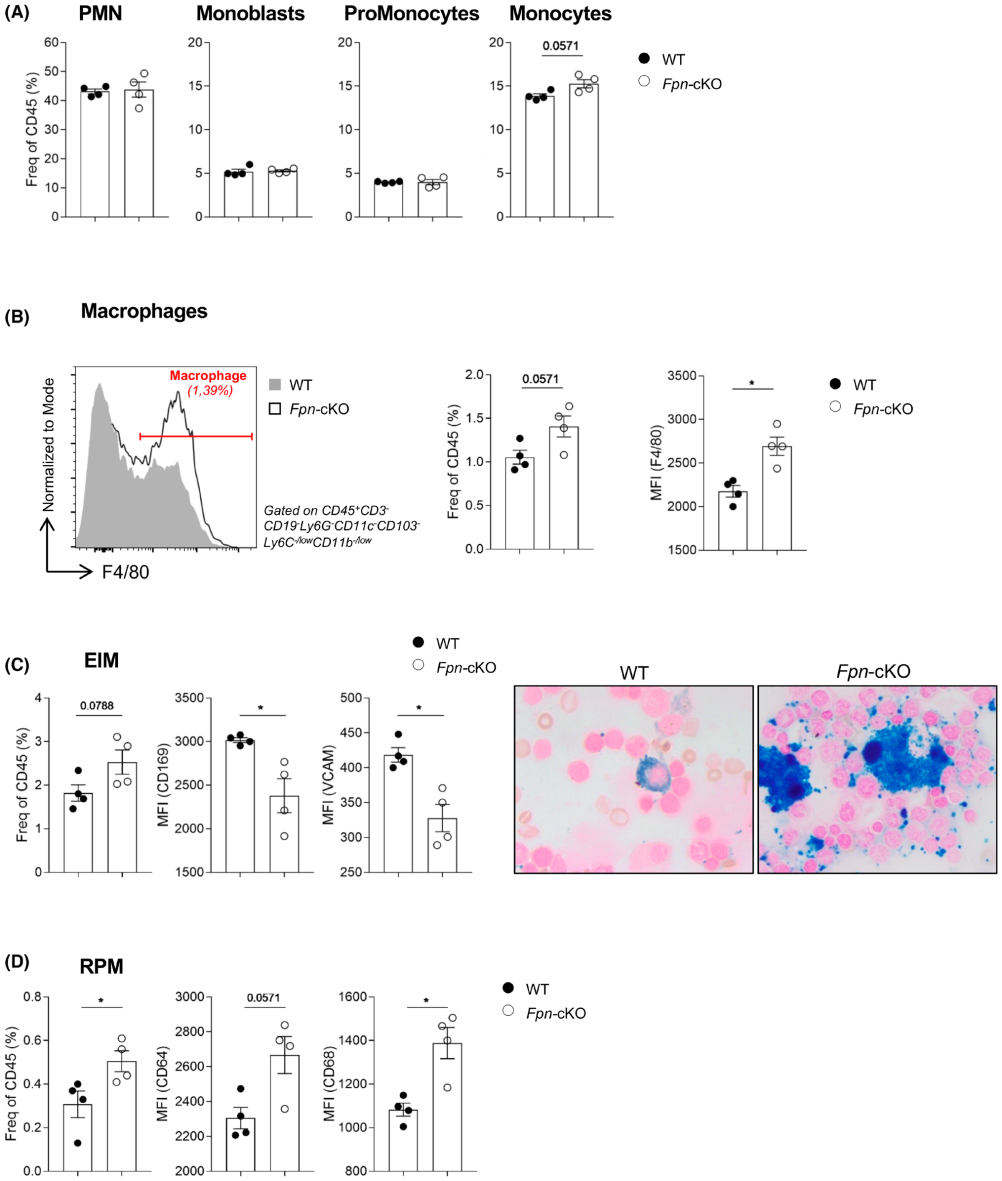
Alterations in bone marrow (BM) myeloid cells of *Fpn*‐cKO mice. (A) Frequencies within BM CD45^+^ cells of polymorphonucleated (PMN) cells, monoblasts, pro‐monocytes and monocytes. (B) Left: Representative histogram overlay of macrophages from WT and *Fpn*‐cKO mice; centre: frequency of macrophages within BM CD45^+^ cells; right: histogram indicating F4/80 expression in macrophages measured as MFI. (C) FACS analysis of erythroblastic island macrophages (EIM) defined as CD45^+^CD3^−^CD19^−^B220^−^NK1.1^−^Ly6G^−^, CD11b^−/low^, F4/80^+^, CD169^+^, CD106 (VCAM1)^+^. From left to right: EIM frequency within BM CD45^+^ cells, expression level of VCAM, surface expression of CD169, representative Perls' Prussian Blue iron staining of BM films in WT and *Fpn*‐cKO mice (magnification: 100×); BM cells were harvested from one lower limb. (D) FACS analysis of red pulp macrophages (RPM) defined as CD45^+^CD3^−^CD19^−^B220^−^NK1.1^−^Ly6G^−^, CD11c^−^, MHCII^+^, strong autofluorescence, Ly6C^−/low^, CD11b^−/low^, F4/80^high^, Tim4^+^, MERTK^+^, CD64^+^, CD68^+^ (see also Figure S4C). From left to right: RPM frequency within spleen CD45^+^ cells, surface expression level of CD64, expression of CD68. Data are presented as mean ± SD. *p < 0.05.

Despite the absolute number of some myeloid precursors was reduced in the BM of *Fpn*‐cKO compared to WT mice (Figure S3A,B), their frequency within CD45^+^ cells was the same, with the exception of monocytes and macrophages that were modestly elevated in the absence of FPN (Figure 4A,B). In addition, *Fpn*‐cKO mice macrophages presented elevated expression of the F4/80 adhesion marker, known to be expressed by EIM and niche macrophages.^7^ Indeed, by flow cytometry, we were able to detect BM EIM, which expressed significantly lower levels of the VCAM and SIGLEC1 (CD169) defining markers and were engulfed with iron (Figure 4C). Last, in the spleen, we found an increased frequency of red pulp macrophages (RPM), which expressed higher levels of surface CD64 and CD68 (Figure 4D; Figure S4C).

### The absence of iron released by myeloid cells is sensed at the apex of the haematopoietic hierarchy

To further investigate the origin of peripheral and medullary erythroid cell alterations, we analysed by FACS all the BM populations responsible for red cell production, including stem cells.

**FIGURE 5:**
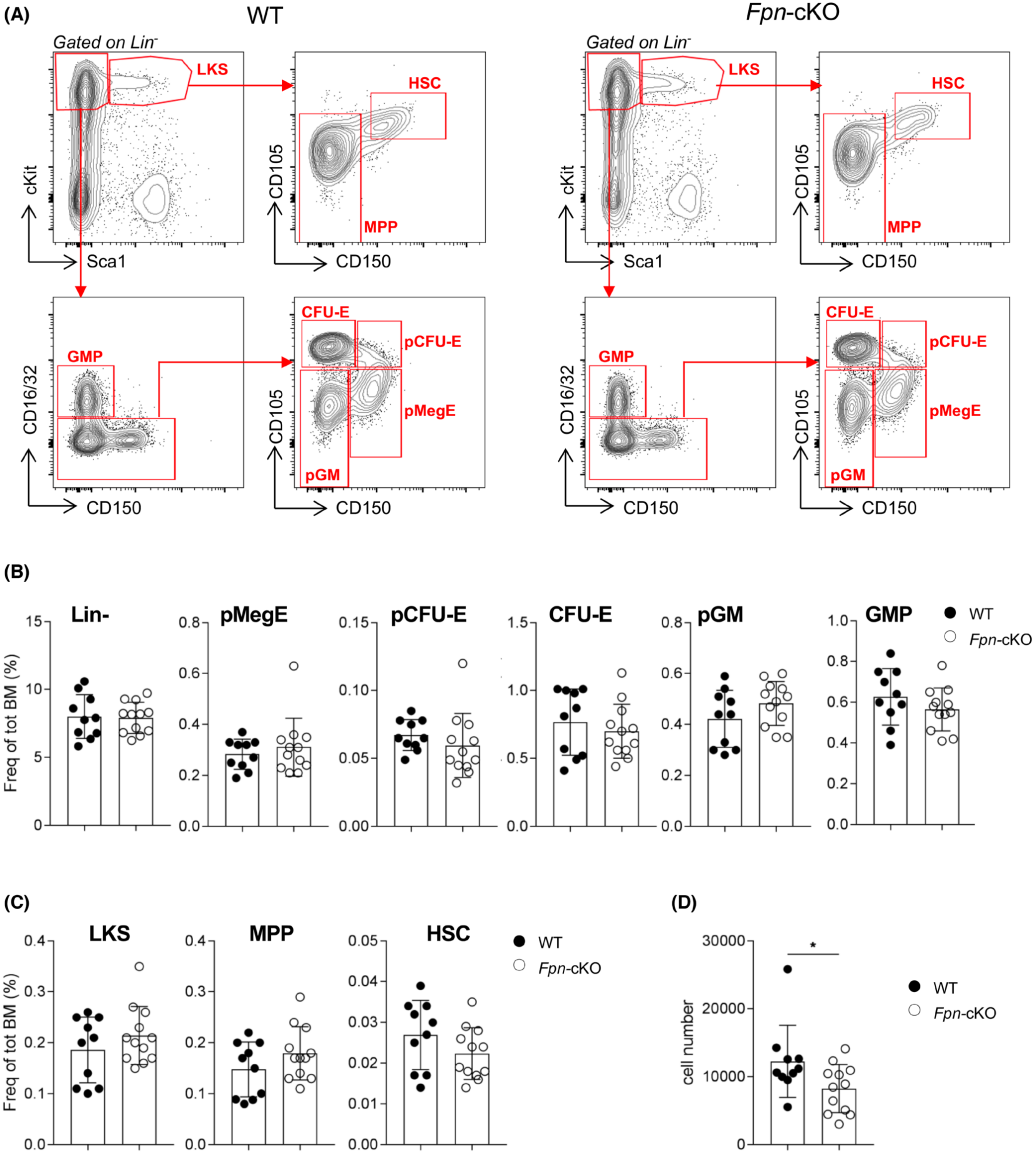
The absence of iron released by myeloid cells is sensed at the apex of the haematopoietic hierarchy. (A) Representative FACS analysis of bone marrow (BM) stem cells, progenitors and precursors. (D) Frequencies of lineage‐negative (Lin^−^) cells, pre‐megakaryocyte‐erythroid progenitor (pMegE) cells, pre‐colony‐forming unit‐erythroid (pCFU‐E) cells, CFU‐E, pre‐granulocyte–macrophage progenitor (pGM) cells and granulocyte–macrophage progenitors (GMP) within total BM. (C) Frequencies of Lin^−^cKit^+^Sca1^+^ (LKS) cells, multipotent progenitor (MPP) cells and haematopoietic stem cells (HSC) within total BM. (D) Absolute number of HSCs within total BM. For all histograms, WT: *n* = 10; *Fpn*‐cKO: *n* = 12. BM cells were harvested from one lower limb. Data are presented as mean ± SD. *p < 0.05.

No alterations in the frequency of lineage‐negative (Lin^−^) early haematopoietic progenitors and of individual Mk/erythrocyte/granulocyte‐macrophage progenitors included in the Lin^−^ cell fraction were present in the BM of *Fpn*‐cKO mice compared to their littermate controls. Similarly, no alteration in the frequencies of upstream Lin^−^cKit^+^Sca1^+^ (LKS) stem and progenitor cells, which include multi‐potent progenitors and HSCs, was detected (Figure 5A–C). However, *Fpn*‐cKO HSCs were reduced in numbers (Figure 5D).

Hence, early haematopoietic stem and progenitor cells, as well as red and Mk lineage committed precursors, are preserved in steady‐state haematopoiesis in the presence of a local iron deficiency, at least in terms of relative frequencies. Nevertheless, a reduction in HSC absolute number was observed, suggesting that the absence of iron released by myeloid cells impinges on the earliest stages of the haematopoietic hierarchy, likely accounting for the observed reduction in the absolute number of BM cells.

### Long‐term deprivation of myeloid‐derived iron triggers local and systemic compensatory mechanisms

**FIGURE 6:**
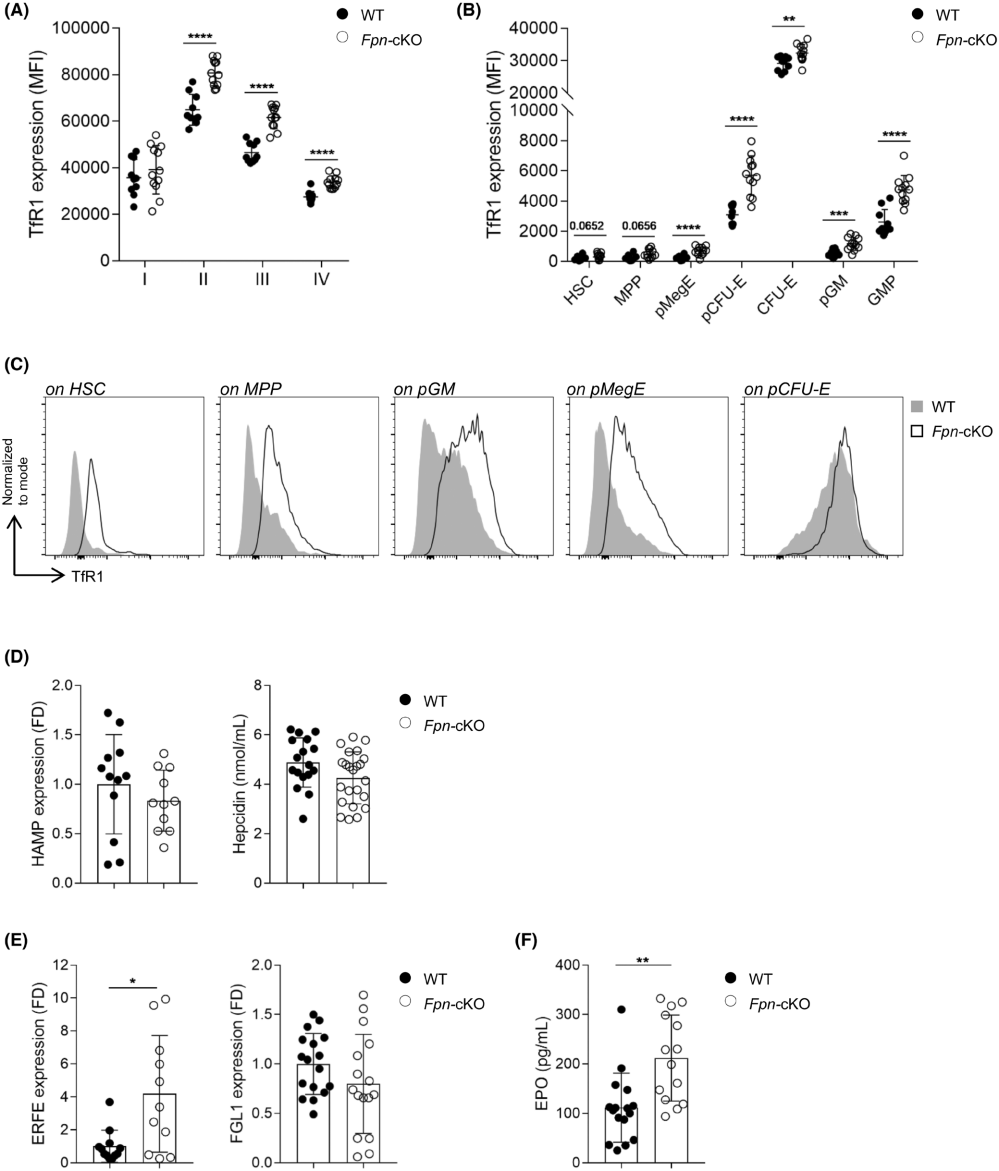
Local and systemic compensatory mechanisms to counteract long‐term deprivation of myeloid‐derived iron. (A) Expression of TFR1 within the different fractions of bone marrow (BM) erythroid development (from I to IV) measured as mean fluorescence intensity (MFI) with a fluorescent‐conjugated anti‐TFR1 monoclonal antibody. (B) Expression of TFR1 within haematopoietic stem cell (HSC) and different progenitors and precursors measured as MFI. (C) Representative histogram overlays indicating the expression of TFR1 as MFI in some of the populations shown in B. (D) Hepcidin mRNA level in liver (HAMP, left) and hepcidin serum concentration (right). (E) ERFE (Fam132b, left) and FGL1 (right) mRNA levels in spleen and liver respectively. All gene expression analyses, referred to 24‐week‐old mice, are measured by quantitative RT‐PCR and normalized to the housekeeping gene 18S RNA or GAPDH. (F) Erythropoietin serum concentration measured by enzyme linked immuno sorbent assay (ELISA) (A, B) WT: *n* = 10; *Fpn*‐cKO: *n* = 12. (D–F) WT: *n* = 12–17; *Fpn*‐cKO: *n* = 11–23. Data are presented as mean ± SD. *p < 0.05; **p < 0.01; ***p < 0.001; ****p < 0.0001.

Since iron homeostasis is essential for the maintenance of proper BM functionality, several compensatory mechanisms could take place to balance the long‐term local iron deficiency observed in *Fpn*‐cKO mice. Along the BM red cell development, a persistent increase in the surface expression of the iron importer Transferrin Receptor 1 (TFR1) was observed in *Fpn*‐cKO mice (Figure 6A). This finding is indicative of increased iron avidity of these highly proliferating cells and in line with the key role of TfR1 in erythroid commitment.^12^ Moreover, TFR1 membrane expression was higher in virtually all the BM subpopulations analysed in *Fpn*‐cKO mice (Figure 6B,C), probably as the result of a massive attempt to overcome the local iron deficiency. This also occurs in cells with apparently no perturbation in frequencies and less iron dependence, including HSCs, known to have little or no membrane expression of TFR1 at steady state to preserve their quiescence,^13^ and to upregulate it for allowing their activation.^12^

In addition to the local BM response, we investigated possible alterations in systemic iron regulation. The hepatic expression of the hepcidin antimicrobial peptide gene (*Hamp*, coding for hepcidin) and the hepcidin serum level were similar in *Fpn*‐cKO and WT mice. Likewise, the hepatic expression of Fibrinogen Like 1 (*FGL1*), one of the hepcidin regulators, was comparable in WT and in *Fpn*‐cKO mice. Conversely, the expression of splenic *Erfe*, the erythroid regulator of iron homeostasis, was increased in *Fpn*‐cKO mice (Figure 6D,E), in line with the sustained extramedullary erythropoietic drive, supported by increased serum erythropoietin (Figure 6F). Therefore, the erythropoietin‐dependent increase of ERFE was insufficient to repress hepcidin, possibly because hepatic iron overloading counters the effect of ERFE.^14^ Given the multiple levels of regulation of hepcidin, of which we evaluated only the FGL1‐dependent one, the lack of hepcidin downmodulation in the presence of increased ERFE levels is not uncommon.^15^

Therefore, though several compensatory mechanisms take place to ensure adequate iron levels, they are not sufficient to fully overcome the anaemia observed in ageing *Fpn*‐cKO mice.

## DISCUSSION

In this report, we show that targeted *Fpn* deletion in myeloid cells results in a functional iron‐deficient anaemia with ageing, characterized by iron trapping within macrophages and impaired iron supply to BM. The low iron availability resulting from tissue macrophages iron sequestration directly correlated to serum ferritin increase^16^ and low total iron binding capacity, a condition similar to ACD.^17^ Different compensatory mechanisms are established, including extramedullary haematopoiesis, erythropoietin‐dependent increased expression of ERFE and increased membrane expression of TFR1.

Despite these attempts, anaemia is not fully overcome. Defects in the haematopoietic system include hypocellular BM with fewer erythroblasts, suggesting that iron release from macrophages plays a role in medullary cell proliferation. This idea is confirmed by the finding that TFR1 surface expression, which indicates low intracellular iron levels, is increased in BM cells. However, we cannot exclude the possibility that the lower number of erythroblasts is instead also due to an accelerated erythrocyte production rate, not sufficiently counterbalanced by a proper erythroid regeneration from earlier progenitors. In either case, homeostasis is not maintained.

We also observed unbalanced erythropoiesis/thrombopoiesis. These findings remind the thrombocytosis typical of mild but chronic iron deficiency in humans^18^ and are in line with the preferential megakaryocytic commitment of megakaryocytic‐erythroid progenitors found in mouse models of iron deficiency.^19^ The regulatory mechanism at the basis of the preferential skewing towards the production of platelets instead of RBC in iron deficiency remains to be fully explained, although a role for the TFR2/ERK pathway has been proposed.^19^ Our results suggest a role for rapid release within the BM, similar to what is demonstrated in the skin during wound healing.^5^ This response may preserve iron for functions other than erythroid differentiation and help stop a life‐threatening acute haemorrhage, conferring a selective advantage. On the other hand, a higher number of platelets may increase the risk of vascular thrombosis that can contribute to augment the age‐related cardiovascular risk. Mk‐biased HSCs are known to expand and grant preferential platelet skewing with ageing,^20^ but also iron overload was shown to lead to HSC bias towards Mk, at least at the transcriptional level.^10^ Therefore, a correct iron balance must be maintained in this HSC subpopulation, while other HSC subsets might be less sensitive to small alterations of iron availability.

We also showed that the local deficiency of iron released by myeloid cells impacts virtually all the cell types involved in RBC development, starting from HSCs, triggering iron avidity through increased TFR1 expression, but without perturbing their frequencies. Upregulation of TFR1 in response to low intracellular iron is a known phenomenon activated by developing cells with high iron demand like erythroid precursors.^21^ We here show that the mechanism is much broader, involving cell development throughout the haematopoietic system also beyond erythroid cells, in accordance with the essential role of iron for haematopoiesis,^22^ as also highlighted by the phenotype of conditional knockout mice with HSC‐specific loss of TFR1.^13^ Increased TFR1 cell surface presentation in HSCs and progenitors has been recently shown in response to acute iron limitation in vitro in response to an iron chelator.^10^ In vivo intermittent treatment with an iron chelator over 1 year increased HSC regenerative capacity, indicating that low iron is indeed beneficial for HSCs,^10^ in line with the known detrimental effects of iron overload in these cells.^22^, ^23^ We did not formally test HSC regenerative capacity through transplantation assays; nevertheless, the occurrence of anaemia with ageing, the HSC bias towards the Mk differentiation and the reduction of HSC and myeloid precursor numbers that we observe upon continuous (not intermittent) lack of iron supply from myeloid cells owing to a genetic defect suggest that having a local source of iron is indeed necessary for proper HSC regeneration. Although most early progenitors display preserved frequencies in *Fpn*‐cKO mice in steady‐state haematopoiesis, their functionality and prompt response could still be affected by peripheral acute stresses, such as bleeding or infections. Moreover, the evolving of other pathological conditions like chronic inflammation or (haematological) cancer could uncover further haematological defects.

In addition to the effects of FPN deletion on neighbouring cells, we also evaluated the impact of iron accumulation in myeloid cells on their own development. We observed an increased frequency of BM macrophages, which displayed higher levels of F4/80. Moreover, specific tissue resident macrophages relevant for correct erythropoiesis, like EIM for BM and RPM in the spleen, were also altered in frequency and in the expression of functional markers. Reduced cell surface expression of FPN was recently documented in macrophages from aged (10‐ to 11‐month‐old) mice, associated with an increased frequency of CD11b^+^F4/80^+^ cells and with increased dysfunction and oxidative stress due to iron accumulation.^24^ Thus, our findings obtained with genetic FPN inactivation are in line with physiological changes occurring during ageing.

In conclusion, FPN‐mediated iron release from myeloid cells is crucial to ensure the homeostasis of the haematopoietic system and to prevent age‐associated anaemia. The high prevalence of anaemia in the elderly is an increasing public health problem that remains largely unexplained^25^, ^26^, ^27^; similarly, iron homeostasis is disrupted in patients with ACD or cancer.^28^ Our study provides preclinical support to further investigate the role of local iron supply in the functions of all players regulating erythropoiesis/megakaryopoiesis and, more broadly, in haematopoiesis, with important implications in the context of anaemia in the elderly.

## AUTHOR CONTRIBUTIONS

Laura Crisafulli, Margherita Correnti and Elena Gammella conducted experiments, acquired, analysed, interpreted data and wrote the manuscript. Elisa De Camilli, Matteo Brindisi, Eleonora Palagano and Chiara Milanesi conducted experiments. Cristina Sobacchi conducted experiments, oversaw data analysis and edited the manuscript. Gabriele Todisco and Matteo G. Della Porta oversaw data analysis and interpreted data. Gaetano Cairo, Stefania Recalcati and Francesca Ficara designed and directed research, oversaw data analysis and wrote the manuscript. All authors approved the final manuscript.

## FUNDING INFORMATION

This work was supported by grants from Ministero dell'Università e della Ricerca to Stefania Recalcati. Francesca Ficara was supported by a Fondazione Cariplo and Telethon Joint grant (GJC21072, project 2022‐0576).

## CONFLICT OF INTEREST STATEMENT

The authors have no conflicts of interest to declare.

## ETHICS STATEMENT

The study was performed in accordance with the guidelines of the Helsinki declaration and approved by the animal welfare office of the Italian Health Ministry (Authorization n° 250/2020‐PR).

## Supporting information


Appendix S1.

## Data Availability

Raw data will be deposited in a public data repository (Zenodo) upon acceptance.
