# Supplementary material for: Iron trapping in macrophages reshapes the homeostasis of the haematopoietic system
Source: Br J Haematol. 2025 Feb 26;206(5):1485–96. doi: 10.1111/bjh.20031 (PMC12078876; doi:10.1111/bjh.20031)
Supplement: Supplementary file 1 — Appendix S1. [file BJH-206-1485-s001.zip › Crisafulli-Correnti_BJH_Suppl Text2-short.docx]

**SUPPLEMENTAL INFORMATION**

**Blood analysis**

Whole blood, withdrawn by cardiac puncture, was collected in K_2_EDTA spray coated microtainer (BD) prior to blood film preparation for standard May-Grünwald Giemsa staining and analysis with an hemocytometer (Mythic18 VET, Orphée). To measure serum iron and ferritin concentration, blood was collected in the absence of anti-coagulant and analyzed with a Ci16200 Architect Abbott instrument (Abbott Park, IL, USA). Transferrin saturation was calculated as serum iron concentration (μg/dL) x 100 / [serum transferrin (mg/dL) x 1.42].

**Histology**

Extramedullary hematopoiesis in the spleen was blindly evaluated semi-quantitatively on 10 fields/section based on the presence of megakaryocytes as follows: 0 = no positive fields; 1 = 1-2 positive fields; 2 = 3-4 positive fields; 3 = > 4 positive fields. For iron detection, sections were stained with Perls’ Prussian blue and the percentage of positive area on total area was quantified with ImageJ software on 10 fields/section as follows: 0 = absence of iron laden areas; 1 = rare iron laden areas (0-10% for spleens; 0-1% for livers); 2 = small number of iron laden areas (10-25% for spleens; 1-3% for livers); 3 = moderate number of iron laden areas (25-35% for spleens; 3-5% for livers); 4 = large number of iron laden areas (>35% for spleens; >5% for livers).

**Liver and spleen non-heme iron content**

Approximately 50 mg of liver or spleen tissue was homogenized in RIPA buffer and a 100μl aliquot was used for the measurement. After addition of 750μl of water and 50μl of 1.5M sodium hydrosulfite the samples were incubated for 1h at 100°C. The samples were then centrifuged at 3000xg, the supernatants transferred in a cuvette and read at 520nm. After addition of 10μl of 5% 2,2’-Bipyridyl in 60% acetic acid, the specific absorbance at 520 nm was read again. A standard curve was obtained from a 5mM stock solution of iron nitrilotriacetate (FeNTA) prepared in water, and the values were calculated after subtraction of the background absorbance.

**Presence of iron in the BM**

BM smears were prepared by push slide technique of BM cells harvested by centrifugation^1,2^. In brief, edges of one lower limb bones (one femur and one tibia) were cut, bones were transferred into one 0,5ml nest tube, then into one 1,5ml tube and centrifuged at 15.000g for 30seconds. Pellet of BM cells was then quickly resuspended with its own plasma and one drop (5-10μl) was smeared onto histological glass slide. Cells were fixed in methanol for 15 minutes at room temperature, air-dried, and submitted for using iron (Perls’ Prussian Blue) staining. Brightfield micrographs were acquired using a Panoramic Olympus camera (DP27) at 100X magnification.

**Flow Cytometry**

BM cells were harvested by flushing of one lower limb (femur and tibia) in FACS buffer (PBS pH 7.2, 2% FBS, 1mM EDTA). Dead cell discrimination was performed with LIVE/DEAD Fixable Aqua Dead Cell Stain Kit (ThermoFisher Scientific), following manufacturer’s instructions. For splenocyte staining, spleens were smashed to recover hematopoietic cells, RBC were lysed, and the remaining cell suspension was incubated with the appropriate cocktail of antibody. Cell staining of BM and spleen cells was performed in FACS buffer using combinations of fluorochrome-conjugated monoclonal antibodies. For myeloid subpopulation analyses BM and spleen cells were incubated for 10 minutes at room temperature with an anti-FcγRII/IIIR blocking antibody prior to incubation with fluorochrome-conjugated antibodies. Data were acquired using the LSR Fortessa or the FACSymphony A5 SE flow cytometers (BD Bioscience) equipped with BD FACSDIVA™ software (BD) and analyzed using FlowJo Software (Tree Star). For sorting of BM erythroblasts live CD11b^-^Ter119^+^CD44^+^ BM cells of individual mice were sorted using FACSAria III sorter. Sorted cells were immediately processed for RNA extraction and gene expression analysis.

All the analyzed subpopulations, and antibodies used to identify them, are listed in Supplemental Tables S1-2.

**Quantitative real-time polymerase chain reaction (qRT-PCR)**

Total RNA was isolated from liver and spleen using TRI reagent® (Sigma-Aldrich). To evaluate expression levels of Erfe and Hamp, reverse transcribed into cDNA with Proto Script M-MuLV First Strand cDNA Synthesis Kit (New England Biolabs, Euroclone), and the obtained cDNA used as a template for Real-Time PCR, based on the TaqMan methodology (Life Technologies, Thermo Fisher). Thermal cycling parameters were 40 cycles at 95° C for 15 s and 60° C for 1 min. Each sample was amplified in triplicate using the primers listed in Supplemental Table S3 (Applied Biosystems, Thermo Fisher). The amount of RNA was calculated using the 2^-ΔCt^ method and results were normalized to 18S rRNA. To evaluate expression levels of FGL1, RNA was reverse transcribed into cDNA with High-Capacity cDNA Reverse Transcription Kit (Applied Biosystems, Thermofisher Scientific), and the obtained cDNA used as a template for Real-Time PCR with SYBR Green gene-specific primer pairs. Changes in the mRNA expression level of target genes were detected using FAST SYBR-Green PCR Master Mix (Applied Biosystems, Thermofisher Scientific). Thermal cycling parameters were 95°C for 20 s and 40 cycles of 3 s at 95° C followed by 30 s at 60° C. Each sample was amplified in triplicate using the primers listed in Supplemental Table S4 (Sigma-Aldrich). The amount of RNA was calculated using the 2^-ΔCt^ method and results were normalized to glyceraldehyde 3-phosphate dehydrogenase (GAPDH). To evaluate expression levels of globin genes, RNA was extracted from freshly sorted BM erythroblast using Direct-zol RNA Microprep kit (Zymo Research), reverse transcribed into cDNA with iScript™ Reverse Transcription Supermix, (Biorad) and 2ng of cDNA was used as a template for Real-Time PCR with SYBR Green gene-specific primer pairs and SsoAdvanced Universal SYBR Green Supermix. (Biorad). Thermal cycling protocol followed manufacturer instructions. Each sample was amplified in triplicate using the primers listed in Supplemental Table S4 (Sigma-Aldrich). The amount of RNA was calculated using the 2^-ΔCt^ method and results were normalized to 18S rRNA.

**ELISA assay**

Serum samples were used to measure the levels of erythropoietin and hepcidin. Erythropoietin levels were measured in accordance with the manufacturer’s instructions (R&D Systems, Space). Hepcidin levels were measured using a specific kit (Intrinsic Lifescience) according to the manufacturer’s instructions.

**Supplemental Table S1**. Fluorochrome conjugated antibodies, related to all figures.

|  | **CLONE** | **FLUOROCHROME** | **SUPPLIER** | **CAT no.** |
| --- | --- | --- | --- | --- |
| CD48 | HM48.1 | PE-Cy7 | eBioscience™ | 25-0481-80 |
| CD117 (cKit) | 2B8 | Super Bright780^TM^ | eBioscience™ | 78-1171-82 |
| CD150 | TC15-12F12.2 | BV605^TM^ | BioLegend | 115927 |
| Ly-6A/E (Sca-1) | D7 | APC | eBioscience™ | 17-5981-82 |
| Lineage cocktail (CD3, B220 CD11b, TER-119,  Gr-1) | 17A2; RA3-6B2;  M1/70;  TER-119; RB6-8C5 | eFluor450 | eBioscience™ | 88-7772-72 |
| CD11b | M1/70 | APC | eBioscience™ | 17-0112-82 |
| CD11b | M1/70 | eFluor450 | eBioscience™ | 48-0112-82 |
| CD16/CD32 | 93 | PerCP-Cy5.5 | eBioscience™ | 45-0161-82 |
| CD16/CD32 | 93 | purified | eBioscience™ | 14-0161-85 |
| CD105 | MJ7/18 | PE | eBioscience™ | 12-1051-82 |
| CD71 | RI7217 | FITC | BioLegend | 113806 |
| CD31 | 390 | PE | eBioscience™ | 12-0311-83 |
| Ly-6C | AL-21 | FITC | BD | 553104 |
| Ly-6G | 1A8 | PE-CF594 | BD | 562700 |
| F4/80 | BM8 | PE-Cy7 | eBioscience™ | 25-4801-82 |
| CD19 | eBio 1D3 | PerCP-Cy5.5 | eBioscience™ | 45-0193 |
| CD3 | 17A2 | BUV496 | BD | 741117 |
| TER119 | TER-119 | PE-Cy7 | eBioscience™ | 25-5921-82 |
| CD44 | IM7 | APC | eBioscience™ | 17-0441-82 |
| CD45 | 30-F11 | BUV805 | BD | 748370 |
| CD64 | X54-5/7.1 | BV650 | BD | 740622 |
| CD103 | M290 | BUV395 | BD | 740238 |
| MHCII | 2G9 | BV711 | BD | 743874 |
| CD11c | HL3 | AF700 | BD | 560583 |

**Supplemental Table S2.** Analyzed BM cell populations

|  | **CELL POPULATION** | **ACRONYM OR ABBREVIATION** | **PHENOTYPE** |
| --- | --- | --- | --- |
| Stem and progenitor cells | Hematopoietic Stem and Progenitor Cells | LSK | Lin^-^/c-Kit^hi^/Sca1^hi^ |
|  | Hematopoietic Stem Cells | HSCs | LSK/CD150^+^/CD105^+^ ^(1)^ |
|  | Multi-Potent Progenitors | MPPs | LKS/CD150^-^/CD105^-^ ^(1)^ |
|  | Granulocyte-  Macrophage Progenitors | GMPs | Lin^-^/c-Kit^+^/Sca1^-^/CD150^-^/CD16/32^+(1)^ |
| Megakaryocyte and  erythrocyte precursors | Pre-MegE | pMegE | Lin^-^/c-Kit^+^/Sca1^-^/CD16/32^-^/CD105^-^/CD150^+(1)^ |
|  | Pre-CFU-E | pCFU-E | Lin^-^/c-Kit^+^/Sca1^-^/CD16/32^-^/CD105^+^/CD150^+(1)^ |
|  | CFU-E |  | Lin^-^/c-Kit^+^/Sca1^-^/CD16/32^-^/CD105^+^/CD150^-(1)^ |
|  | Pre-GM | pGM | Lin^-^/c-Kit^+^/Sca1^-^/CD16/32^-^/CD105^-^/CD150^+(1)^ |
|  | Erythrocyte precursors (at different stages of maturation) | From stage I to stage V ^(2)^ | Ter119^-^/CD44^high^ (I); Ter119+/CD44^high^ or CD44^int^ or  CD44 ^low^ or CD44^-^ (II to V) |
| Myeloid precursors and mature cells  *(all gated on CD45^+^ cells)* | Poly-Morpho-Nucleated Leukocytes | PMN | Not Lympho (CD3^-^/CD19^-^)/  Not Dendridic (CD11c^-^/CD103^-^/MHCII^low/+^)/ CD11b^+^/Ly6G^high^ |
|  | Monoblasts |  | Not Lympho (CD3^-^/CD19^-^)/  Not Dendridic (CD11c^-^/CD103^-^/MHCII^low/+^)/ not PMN (Ly6G^-^)/CD31^+^/Ly6C^-^ |
|  | Pro-Monocyte |  | Not Lympho (CD3^-^/CD19^-^)/  Not Dendridic (CD11c^-^/CD103^-^/MHCII^low/+^)/ not PMN (Ly6G^-^)/CD31^+^/Ly6C^+^ |
|  | Monocyte |  | Not Lympho (CD3^-^/CD19^-^)/  Not Dendridic (CD11c^-^/CD103^-^/MHCII^low/+^)/Not PMN/ (Ly6G^-^)/CD11b^+^/CD31^-^/Ly6C^+^ |
|  | Macrophage |  | Not Lympho (CD3^-^/CD19^-^)/  Not Dendridic (CD11c^-^/CD103^-^/MHCII^low/+^)/ Not PMN (Ly6G^-^)/Ly6C^-/low^)/CD11b^+/low^/F4/80^+^ |
|  | Erythroblastic Island macrophages | EIM^(3)^ | Not Lympho (CD3^-^/B220^-^/CD19^-/^NK1.1^-^)/ Ly6G^-^/CD11b^-/low^/F4/80^+^/CD169^+^/CD106 (VCAM1)^+^ |
|  | Red Pulp Macrophages | RPM^(4)^ | Not Lympho (CD3^-^/B220^-^/CD19^-/^NK1.1^-^)/ Ly6G^-^/CD11c^-^ /MHCII^+^/Strong Autofluorescence/Ly6C^-/low^/CD11b ^-/low^/F4/80^high^, Tim4^+^/MERTK^+^/CD64^+^/CD68^+^ |

(1) Modified from Pronk CJH. et al.^3^; (2) Modified from Liu J. et al.^4^; (3) Modified from Seu KG. et al.^5^; (4) Modified from Fujiyama S. et al.^6^

**Supplemental Table S3.** Primers for TaqMan qRT-PCR.

| **Gene** | **TaqMan gene expression assay ID** | **Supplier** |
| --- | --- | --- |
| Rn18s | Mm03928990_g1 | Applied Biosystems, Thermo Fisher |
| Fam132b | Mm00557748_m1 | Applied Biosystems, Thermo Fisher |
| Hamp | Mm04231240_s1 | Applied Biosystems, Thermo Fisher |

**Supplemental Table S4.** Primers for SYBR Green qRT-PCR.

| **Gene** | **Primer Forward** | **Primer Reverse** | **Supplier** |
| --- | --- | --- | --- |
| mGAPDH | CTGCCACCCAGAAGACTGTG | GGTCCTCAGTGTAGCCCAAG | Sigma-Aldrich |
| mFGL1 | CGATCTGATGGCAGTGAGAACT | TTTGTTACCCAGCCAGTATTCG | Sigma-Aldrich |
| m_Hba | GCTGAAGCCCTGGAAAGGAT | CAGAGCCGTGGCTTACATCA | Sigma-Aldrich |
| m_Hbb | GCTGCATGTGGATCCTGAGA | CTTCTGGAAGGCAGCCTGTG | Sigma-Aldrich |
| 18S rRNA | GTTGCCAAGCCTGGAGATAA | GGTGGCGGATTCTTAGGTTC | Sigma-Aldrich |

REFERENCES

1. Smith AO, Adzraku SY, Ju W, Qiao J, Xu K, Zeng L. A novel strategy for isolation of mice bone marrow endothelial cells (BMECs). *Stem Cell Res Ther*. May 03 2021;12(1):267. doi:10.1186/s13287-021-02352-3

2. Yue Z, Niu X, Yuan Z, et al. RSPO2 and RANKL signal through LGR4 to regulate osteoclastic premetastatic niche formation and bone metastasis. *J Clin Invest*. Jan 18 2022;132(2)doi:10.1172/JCI144579

3. Pronk CJ, Rossi DJ, Månsson R, et al. Elucidation of the phenotypic, functional, and molecular topography of a myeloerythroid progenitor cell hierarchy. *Cell Stem Cell*. Oct 2007;1(4):428-42. doi:10.1016/j.stem.2007.07.005

4. Liu J, Zhang J, Ginzburg Y, et al. Quantitative analysis of murine terminal erythroid differentiation in vivo: novel method to study normal and disordered erythropoiesis. *Blood*. Feb 21 2013;121(8):e43-9. doi:10.1182/blood-2012-09-456079

5. Seu KG, Papoin J, Fessler R, et al. Unraveling Macrophage Heterogeneity in Erythroblastic Islands. *Front Immunol*. 2017;8:1140. doi:10.3389/fimmu.2017.01140

6. Fujiyama S, Nakahashi-Oda C, Abe F, Wang Y, Sato K, Shibuya A. Identification and isolation of splenic tissue-resident macrophage sub-populations by flow cytometry. *Int Immunol*. Feb 06 2019;31(1):51-56. doi:10.1093/intimm/dxy064

**SUPPLEMENTAL FIGURE LEGENDS**

**Figure S1. Cytological and histological analysis of blood and BM from *Fpn*-cKO and WT mice**

(A) Representative images of blood cytology (May-Grunwald-Giemsa staining) showing increased numbers of hypochromic RBC (some examples are indicated with yellow arrows); two reticulocytes are indicated with black asterisks in the representative WT sample. Brightfield micrographs were acquired using a Panoramic Olympus camera (DP27) at 100X magnification. (B) BM histology (H&E staining) showing increased number of megakaryocytes (indicated with yellow arrowheads) in *Fpn*-cKO mice compared to littermate WT controls (top: scale bar 100 μm; bottom: higher magnification of the dashed areas indicated in the top images, scale bar 50 μm).

**Figure S2. Additional blood analysis of *Fpn*-cKO and WT mice**

Additional hemocytometric parameters relative to Total White Blood Cell (WBC) counts (left); counts and percentages (%) of the different WBC subpopulations (Lymphocytes, Monocytes, Granulocytes). For all histograms, WT: n=10; *Fpn*-cKO: n=12. Data are presented as mean ± SD.

**Figure S3. Analysis of myeloid cell subpopulation in *Fpn*-cKO and WT mice**

(A) Gating strategy for the analysis of myeloid cell populations. (B) Number of BM myeloid cell population. Data are presented as mean ± SEM. For PMN, Monoblast, ProMonocyte and Monocyte histograms, WT: n=14; *Fpn*-cKO: n=16. Four of the 14 WT and four of the 16 *Fpn*-cKO samples were stained with an antibody cocktail that included the anti-CD45 and were used to generate Figure 4A-B. For the Macrophage histogram, n=4 *p<0.05. (C) Representative FACS analysis of spleen RPM, showing gating strategy (left) and expression of typical RPM markers (right).
