# Supplementary material for: Iron trapping in macrophages reshapes the homeostasis of the haematopoietic system
Source: Br J Haematol. 2025 Feb 26;206(5):1485–96. doi: 10.1111/bjh.20031 (PMC12078876; doi:10.1111/bjh.20031)
Supplement: Supplementary file 1 — Appendix S1. [file BJH-206-1485-s001.zip › Suppl Figures_BJH_REV_v4.pptx]

## Slide 1
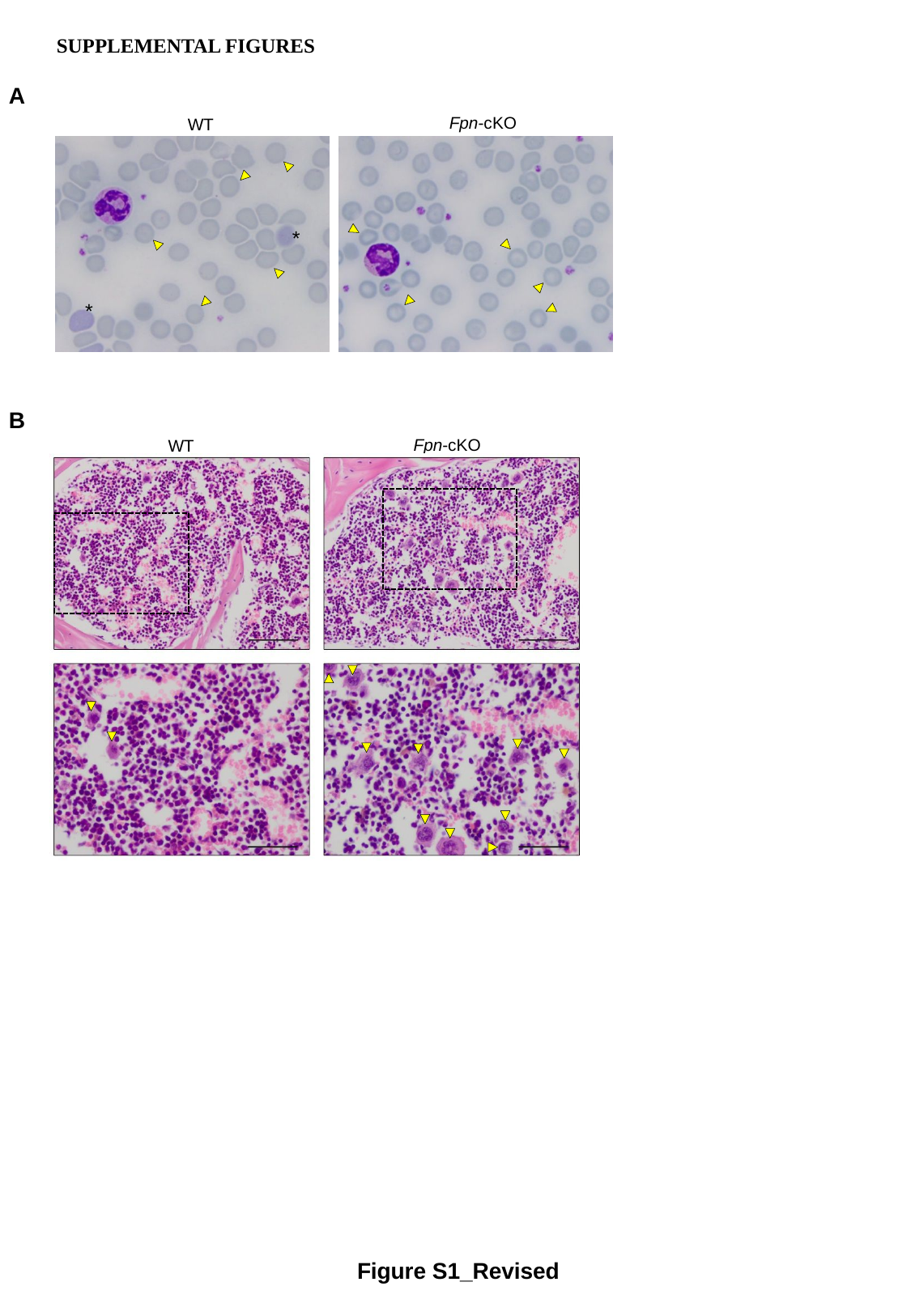

SUPPLEMENTAL FIGURES
A
Fpn-cKO
WT
*
*
B
Fpn-cKO
WT
Figure S1_Revised

## Slide 2
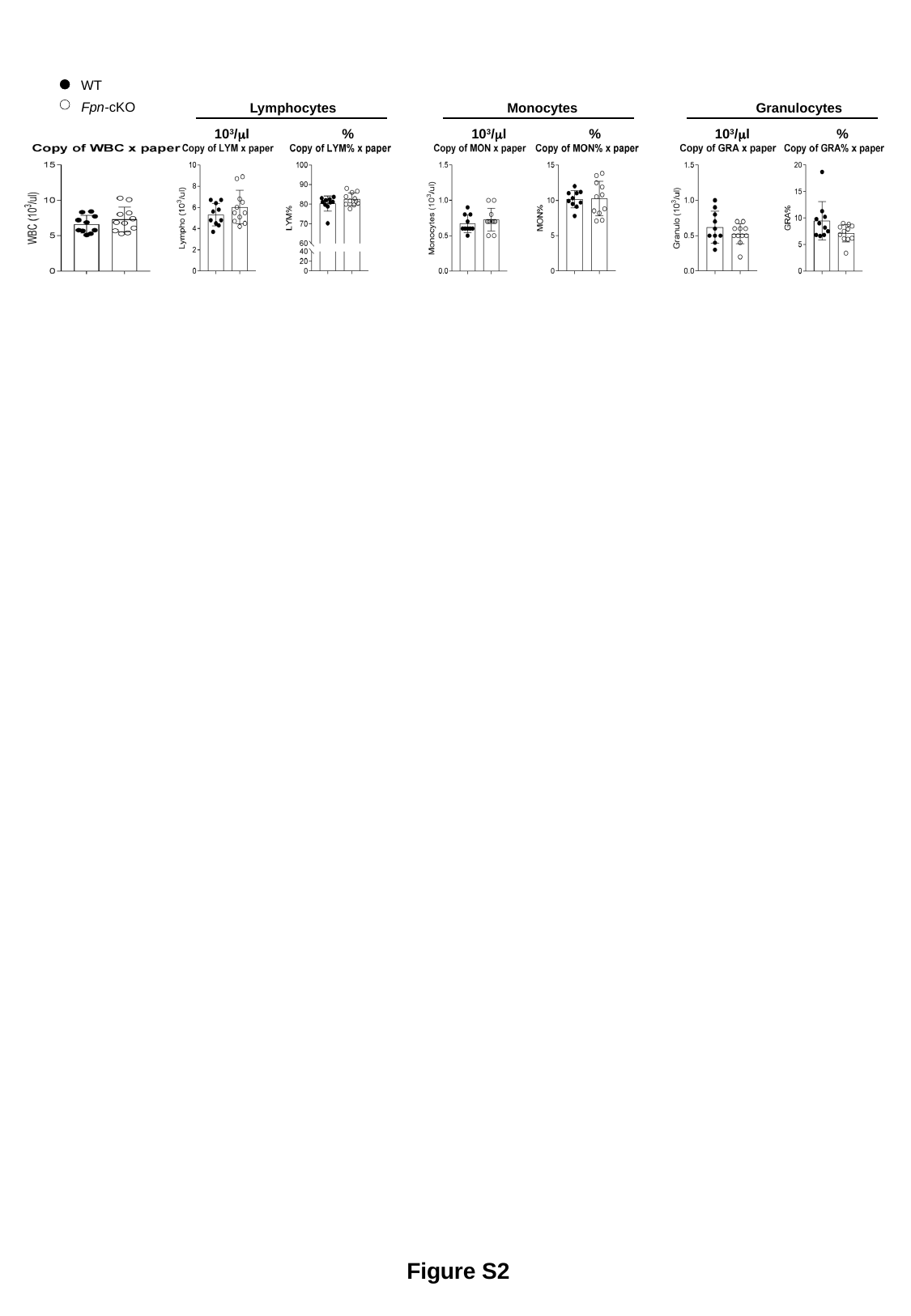

WT
Fpn-cKO
Lymphocytes
Monocytes
Granulocytes
103/ml
%
103/ml
%
103/ml
%
Figure S2

## Slide 3
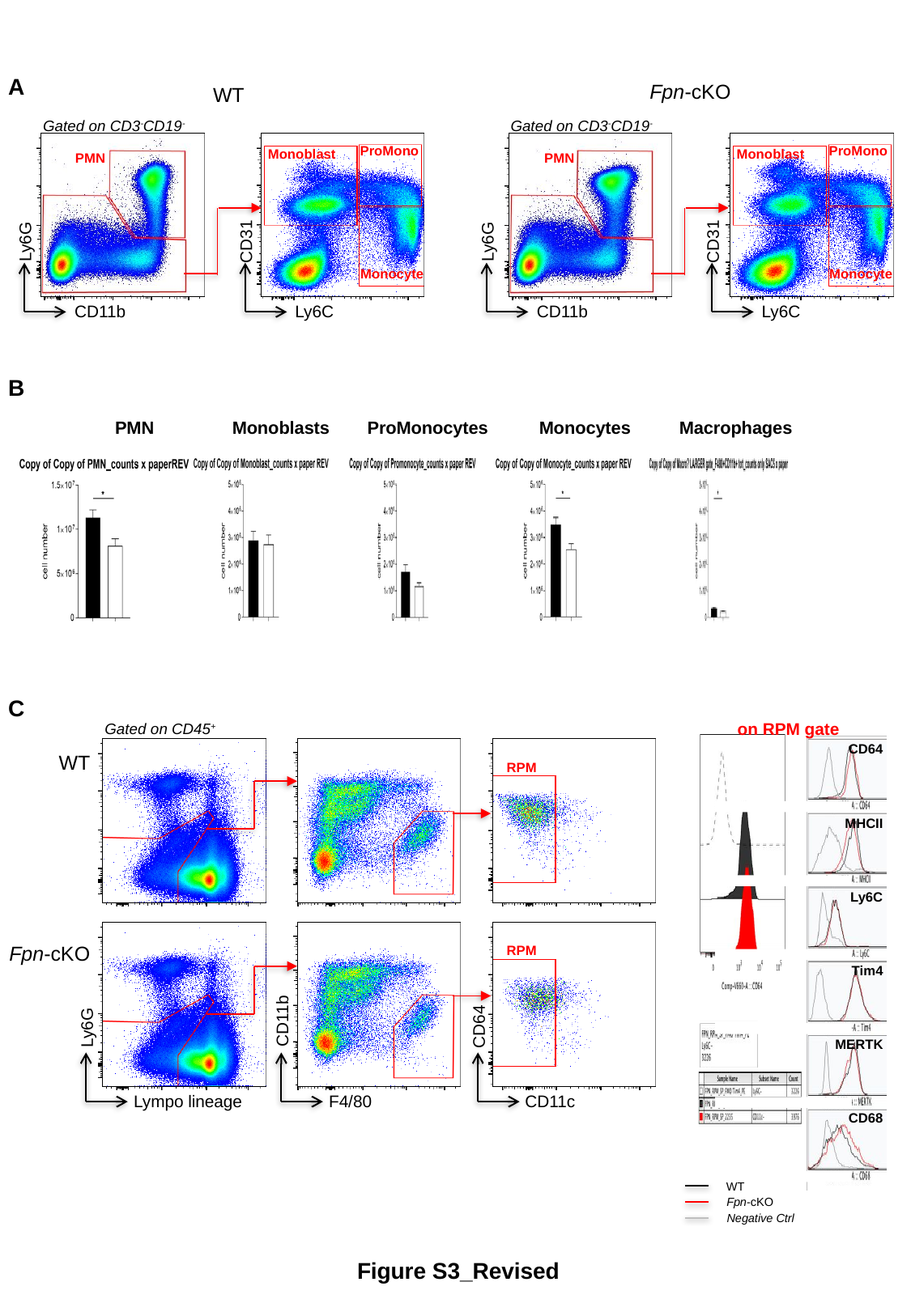

A
Fpn-cKO
WT
Gated on CD3-CD19-
Gated on CD3-CD19-
ProMono
ProMono
Monoblast
Monoblast
PMN
PMN
CD31
Ly6C
CD31
Ly6C
Ly6G
CD11b
Ly6G
CD11b
Monocyte
Monocyte
B
PMN
Monoblasts
ProMonocytes
Monocytes
Macrophages
C
on RPM gate
CD64
MHCII
Ly6C
Tim4
MERTK
CD68
Gated on CD45+
WT
RPM
Fpn-cKO
RPM
CD11b
F4/80
CD64
CD11c
Ly6G
Lympo lineage
WT
Fpn-cKO
Negative Ctrl
Figure S3_Revised
